# Green alternatives to petroleum-based plastics: production of bioplastic from *Pseudomonas neustonica* strain NGB15 using waste carbon source

**DOI:** 10.1007/s11356-024-33309-7

**Published:** 2024-04-16

**Authors:** Nurdan Gönül Baltacı, Mustafa Özkan Baltacı, Arzu Görmez, Serkan Örtücü

**Affiliations:** 1 Department of Molecular Biology and Genetics, Faculty of Science, Ataturk University, 25240 Erzurum, Turkey; 2 Department of Biology, Faculty of Science, Dokuz Eylul University, 35390 Izmir, Turkey; 3 Department of Molecular Biology and Genetics, Faculty of Science, Erzurum Technical University, Erzurum, Turkey

**Keywords:** Biodegradable plastic, Polyhydroxyalkanoates, Cost-effective production, Waste feedstocks

## Abstract

Polyhydroxyalkanoates have attracted great interest as a suitable alternative to petrochemical based plastics due to their outstanding properties such as biodegradability and biocompatibility. However, the biggest problem in the production of microbial polyhydroxyalkanoates is low cost-effectiveness. In this study, polyhydroxyalkanoate production was carried out using waste substrates with local isolates. Culture conditions were optimized to increase the polyhydroxyalkanoate production potential. The produced polyhydroxyalkanoate was characterized by FTIR analyses, and its metabolic pathway was determined by real-time PCR. According to the results, the best polyhydroxyalkanoate producer bacteria was characterized as *Pseudomonas neustonica* NGB15. The optimal culture conditions were detected as 30 g/L banana peel powder, 25 °C temperature, pH 8, and 4-day incubation time. Under the optimized conditions, 3.34 g/L PHA production was achieved. As a result of FTIR analyses, major peaks were obtained at 1723, 1277, 1261, 1097, 1054, and 993 cm^−1^. These peaks represent that the type of produced polyhydroxyalkanoate was poly-β-hydroxybutyrate. According to gene expression profile of NGB15, it was determined that *Pseudomonas neustonica* NGB15 produces PHA using the de novo fatty acid synthesis metabolic pathway. In conclusion, poly-β-hydroxybutyrate production by *Pseudomonas neustonica* NGB15 using a low-cost fermentation medium has been shown to be biotechnologically promising.

## Introduction

Petroleum-based plastics (synthetic plastic), which have wide applications in the household, pharmaceutical, and commercial sectors, have become an important raw material in modern society (Nanda et al. 2022). In 2019, global plastics production was 368 metric tonnes (Mt) and is expected to exceed 600 Mt in 2025 (Tyagi et al. 2021). On the other hand, it is observed that 10 million tons of synthetic plastic leaks into the oceans every year, and this has a harmful effect on the ocean ecosystem (Boucher and Billard 2019). Due to the developing technology and population growth, the demand for plastic and plastic derivative products is constantly increasing (Geyer et al. 2017). This causes serious environmental problems. It is known that petroleum-based plastics cause very serious ecological problems due to their low decomposition rate, the leakage of carcinogenic substances into the environment when exposed to heat, and the formation of toxic by-products in this process (Gironi and Piemonte 2011). There are several solutions to the environmental pollution caused by plastic waste. These are as follows: incineration, recycling practices, or producing and using biodegradable plastics (bioplastics) (Evode et al. 2021). The destruction of petroleum-based plastics by burning and the emergence of harmful gases such as hydrochloric acid and hydrogen cyanide during the process are methods that put the environment and human health at risk (Roy et al. 2021). Although recycling applications are seen as an effective solution, the process is tremendously slow, and the necessity of separating and then recycling plastics with different structures poses a problem for this application (Vogt et al. 2021). In addition to all these, the presence of additives such as fillers and colorants used in plastics limits the recycling of synthetic plastics. In order to reduce the harmful effects of petroleum-based plastics caused by overuse and accumulation, there is a need for environmentally friendly new polymers that are easily degradable and do not harm the environment when decomposed as an alternative to petroleum-based plastics (Naser et al. 2021). Polyhydroxyalkanoates (PHA), known as bioplastics, are seen as an alternative to synthetic plastics due to their physicochemical and mechanical properties (Alcantara et al. 2020). Unlike petroleum-based plastics, bioplastics are becoming popular recently due to their biodegradability, biocompatibility, environmentally friendly production processes, and wide range of applications (Albuquerque and Malafaia 2018).

Polyhydroxyalkonates are produced and stored by bacteria in the absence of essential nutrients such as phosphorus, nitrogen, and sulfur and when there is an excess of carbon source (Kumar et al. 2020). The interest in bioplastics is increasing day by day with the positive results obtained regarding the synthesis and industrial applications of biodegradable environmentally friendly plastics such as PHA by bacteria (Behera et al. 2022). Although bioplastics are environmentally friendly, easily biodegradable, and not harmful to human health, but the production of bioplastics process is more expensive than synthetic plastics (Kumar et al. 2020). The species *Cupriavidus necator* is widely studied for the biosynthesis and production of PHAs (Bellini et al. 2022). Recently, with the emergence of new processes and technologies for the production of economical PHAs, PHA production has been successfully performed in species such as *Bacillus* sp. (Yasin and Al-Mayaly 2021), *Pseudomonas* sp. (Kanavaki et al. 2021), *Aeromonas hydrophila* (Szacherska et al. 2022), *Rhodopseudomonas palustris* (Brown et al. 2022), *Burkholderia sacchari* (Oliveira-Filho et al. 2022), and *Halomonas boliviensis* (Arcila-Echavarría et al. 2022).

Although bioplastics are seen as an alternative to synthetic plastics recently, they need to be produced at a more cost-effective which is one of the major stumbling blocks in the expansion and growth of the PHA market (Koller et al. 2017). Several critical factors are considered important to make bacterial PHA production cost-effective. These:

1. Screening and selection of bacterial strains that have the potential to produce high amounts of PHA and even the discovery of new bacterial strains are required (Kumar et al. 2016)
2. Use of cheap carbon sources in the production process (Zahari et al. 2014)
3. Illuminating PHA synthesis pathways (Możejko-Ciesielska and Mostek 2019a)

The aim of this study is to determine and improve the optimum growing conditions for PHA production by a local strain for possible industrial usage. As a result, PHA production by *P. neustonica* strain NGB15 was evaluated, and its yield was enhanced by the optimization of some culture conditions. Subsequently, in an effort to increase PHA production while lowering fermentation costs, agro-residues including banana and peanut peels were assessed as alternative carbon sources in *P. neustonica* NGB15 fermentation for the first time.

## Materials and methods

### Collection of samples

The most important condition for PHA production in bacteria is the presence of a high carbon source in the culture medium. For this purpose, waste samples from the Erzurum sugar factory, which have a high carbon source content, were brought to the laboratory environment in sterile glass bottles under aseptic conditions for the isolation of PHA-producing bacteria.

### Preparation of substrates

Banana and peanut peels were washed properly, followed by oven drying at 80 °C. The dried banana and peanut peels were ground into fine particles by blender and termed banana peel powder (BPP) and peanut peel powder (PPP) respectively.

### Isolation and screening of PHA-producing bacteria

Sugar factory waste samples were added to the flask containing 100 ml of Tryptic Soy Broth (TSB) and incubated at 25 °C for 48 h. Then, serial dilutions were prepared, and the bacteria were purified (Baltaci et al. 2024). Since PHA production of bacteria absolutely depends on the content of the medium (high carbon source, low sulfur rate, etc.), pure cultured organisms were inoculated into Minimal Davis Broth (MDB) medium, which was used in previous literature data, to stimulate PHA production. Next, the PHA production potential of the bacteria was determined according to the protocol of Taran et al. (2011). The best PHA producer isolate was selected.

### Determination of PHA production

PHA production was determined, using the procedure reported by Taran (2011). Briefly, 5 ml of culture was centrifuged, and the pellets were dissolved in the dH_2_O and incubated at 25 °C for an hour to cells’ total lysis. Then the lysate was centrifuged, and the same volume of ethanol and acetone was added to the pellet. The residue was dissolved in chloroform.

The mixture was kept at room temperature to evaporate the chloroform. After being treated with 5 mL of 17.8 M H_2_SO_4_, the resulting pellet was incubated in boiling water for 40 min. Following this stage, an ultraviolet–visible (UV–Vis) spectrophotometer (Shimadzu, UV 1800 240 V, Japan) was used to measure the generation of crotonic acid at OD235, with H_2_SO4 serving as a blank. Using polyhydroxybutyrate as a reference, the concentration of PHB in the sample was determined (Sigma-Aldrich).

### Molecular characterization of best PHA producer isolate

The Promega WizardR Genomic DNA Purification Kit (A2360) was used for the genomic DNA isolation of the best strain. The 16S rRNA gene region was amplified using 27F (5′-AGAGTTTGATCCTGGCTCAG-3′) and 1492R (5′-GGTTACCTTGTTACGACTT-3′) primers (Baltaci 2022; Baltaci et al. 2020b). Using the pGEM-T Easy Cloning Vector (Promega, Southampton, UK), the PCR product was cloned into the *Escherichia coli* JM101 strain in accordance with the manufacturer’s instructions. The Macrogen Company (Amsterdam, Netherlands) performed the sequence analysis following the cloning (Adiguzel et al. 2019; Akbulut et al. 2022). After the 16S rRNA was acquired, its similarity rate was calculated, and GenBank accession numbers were obtained by comparing it to the other bacterial series found in GenBank and EzTaxon (http://blast.ncbi.nlm.nih and http://www.eztaxon.org). A phylogenetic tree was constructed with Mega4 software based on the 16S rDNA sequences (Baltaci and Adiguzel 2016; Baltaci et al. 2017).

### Optimization of culture conditions

After it was detected the best PHA producer isolate, the culture conditions were optimized. For this, initial optimization experiments were carried out to determine the best waste carbon source and concentration and temperature. Then, pH and incubation time parameters were optimized (Baltaci et al. 2022). Detailed experimental conditions for the optimization studies are given Table 1.

**Table 1:** The experimental conditions for the optimization studies

Waste carbon source	Carbon source concentration (g/L)	Temperature (°C)	pH	Incubation time (day)
Waste baklava syrup	10	15	4	1
Banana peels	20	20	5	2
Peanut peels	30	25	6	3
	40	30	7	4
		35	8	5
		40	9	6
			10	7
			11	

### PHA extraction

NGB15 was grown under optimum culture conditions. Then, the culture was centrifuged, the cells were washed with distilled water, and the cells were lyophilized. Lyophilized cell powder was suspended in 20 mL of chloroform and 20 mL of sodium hypochlorite solution (30%, pH 12.15). The suspended culture was incubated at 30 °C for 150 min and centrifuged at 30 °C for 20 min. After centrifugation, three different phases were formed. The bottom chloroform phase was carefully collected and recovered by PHA (80% methanol) precipitation and filtration (Saratale and Oh 2015).

### ATR-FTIR analysis

The functional groups found in the bioplastics were identified by infrared (IR) spectra. FTIR analysis of the PHA sample was performed at the Eastern Anatolia High Technology Application and Research Center (DAYTAM). The purified PHA sample (2.5 mg) was analyzed by attenuated total reflectance (ATR) technique using a Nicolas iS50 spectrometer (Thermo Fisher Scientific, Waltham, USA). The absorption was scanned at a spectral range of 400–4000 cm^−1^ (Kurt-Kizildogan et al. 2021).

### Determination of metabolic pathway of PHA production

To determine the metabolic pathways through which NGB15 produces PHA under the optimized conditions, the expression of genes involved in these metabolic pathways was determined by real-time PCR. For this purpose, NGB15 was grown under optimized culture conditions, and RNA isolation was performed using the RNeasy Mini Kit (Qiagene-Germany) in accordance with the manufacturer’s instructions. Then, cDNA was synthesized using ThermoScript™ RT-PCR System for First-Strand cDNA Synthesis Kit (Invitrogen-USA). Real-time PCR was performed with Maxima SYBR Green/ROX qPCR Master Mix (2 ×) (Qiagen, Germany) in Rotor-Gene Q 6000 Real-time PCR system (Qiagen, Hilden, Germany). The reaction was carried out in a 20-µL mixture for each tube containing 2 µL cDNA template, 10 µL master mix, 1 µL of each primer, and 6 µL sterilized nuclease-free water. The program was as follows: initial denaturation step at 95 °C for 5 min, followed by 40 cycles of denaturation at 95 °C for 15 s, annealing temperature for each primer for 30 s, and extension at 72 °C for 20 s. Fluorescence signals were measured after the annealing/extension step of each cycle. The primer list of target genes is given in Table 2. 16S rRNA gene was used for normalization and relative quantification.

**Table 2:** Primer sequence of target genes

Gene name	Primer Sequence	Product length (bp)	References
*phaA*	5′GAGAACGTGGCCAAGGAATA-3′	119	(Hiroe et al. 2013)
	5′-GGGACGATCTCTTCGTCAAA-3′		
*phaB*	5′-GATCGACACCAACCTGACCT-3′	102	(Hiroe et al. 2013)
	5′-TTCACCGACGAGATGTTGAC-3′		
*phaC*	5′-GAACGACCTGGTGTGGAACT-3′	150	(Hiroe et al. 2012)
	5′-TCGTTCTGCAGGTAGGTGTG-3′		
*phaJ*	5′- GTTCCGGAGCTTCGTTATG -3′	401	(Reddy et al. 2020)
	5′- GTATTGTCGCTGTTCCTTCC -3′		
*fabG*	5′- TGCATCACCTCTGTTTCAG-3′	207	This study
	5′- CGGGATCATCTTCATCAGTT-3′		
*phaG*	5′-GCCTGTATCCGCAATTCAAC-3′	249	(Wang et al. 2018)
	5′-CCTTCGTCAGCATCTTCTCAT-3′		
*16S rRNA gene*	5′-AGAAGCTTGCTCTTTGCTGA-3′	120	(Hiroe et al. 2012)
	5′-CTTTGGTCTTGCGACGTTAT-3′		

## Result and discussion

### Isolation and screening PHA producer bacteria

A total of 15 bacteria with PHA-producing potential were isolated from waste sugar factory samples, and PHA production amounts were determined spectrophotometrically according to the Taran method (Table 3) (Taran 2011).

**Table 3:** Screening of PHA production potential of isolates

Isolate	PHA production (OD_235_)	PHA yield (g/L)
NGB1	0.09	0
NGB2	0.01	0
NGB3	0.54	0.35
NGB4	0.01	0
NGB5	0.96	1.24
NGB6	0.42	0.1
NGB7	0.13	0
NGB8	1.08	1.50
NGB9	0.88	1.07
NGB10	0.68	0.65
NGB11	1.12	1.58
NGB12	0.36	0
NGB13	0.67	0.63
NGB14	0.49	0.25
**NGB15**	**1.24**	**1.83**
NGB16	0.22	0
NGB17	0.02	0

Bold entries indicate best PHA producer bacteria and PHA production amount

**Fig. 1:**
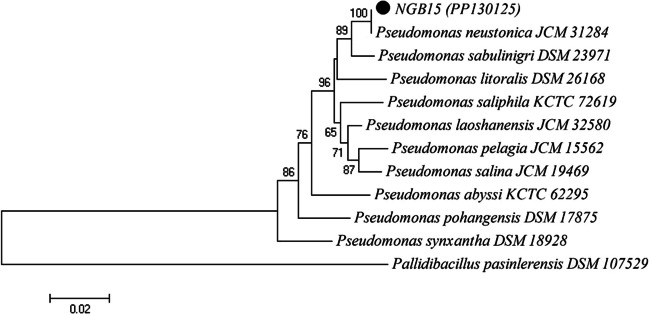
Phylogenetic relationships of NGB15 starin based on 16 S rRNA gene sequence analyses. The tree was constructed by a neighbor-joining method

It was determined that the NGB15 had the highest PHA production potential; therefore, the next experiments were continued with this isolate. Then, molecular characterization of NGB15 was performed by 16S rRNA gene sequence analysis. As a result of 16S rRNA gene sequence analysis, it was determined that NGB15 belonged to *P. neustonica* with a 99.72% similarity rate. Neighbor-joining phylogenetic tree on the basis of 16S rRNA gene sequence data of NGB15 was constructed using the MEGA4 program. *Pallidibacillus pasinlerensis* was used as an out-group (Baltaci et al. 2020a). Bootstrap values based on 1000 replications are listed as percentages at branching points. Only bootstrap values > 50% are shown at nodes. The scale bar represented a 2% divergence (Fig. 1). In the literature, although there are many *Pseudomonas* species producing bioplastic (Manso et al. 2012; Zong et al. 2022), this study is the first study on bioplastic production from *P. neustonica*.

### Optimization of culture conditions

**Fig. 2:**
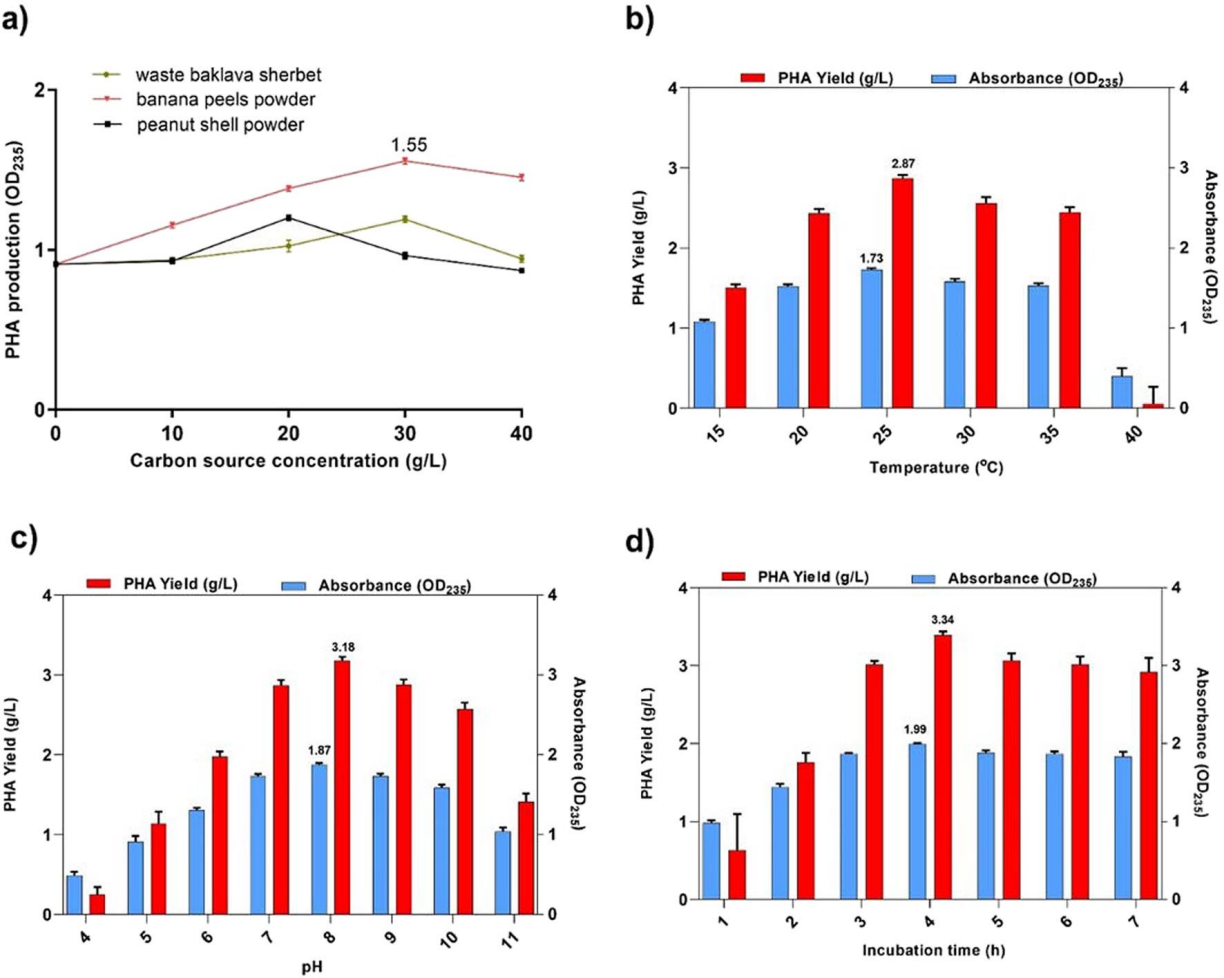
Optimization of culture conditions in PHA production from *P. neustonica* NGB15. **a** Effect of Carbon source and concentration parameters, **b** effect of temperature parameter, **c** effect of pH parameter, **d** effect of Incubation time parameter

Numerous studies have demonstrated that a variety of physicochemical parameters, including type and concentration of nitrogen and carbon sources, cultivation time, pH, temperature, and agitation affect the production of PHA (Anjum et al. 2016; Moreno et al. 2015; Tarrahi et al. 2020). Thus, the one-variable-at-a-time (OVAT) strategy was used to optimize some culture parameters, including substrate concentration, pH, temperature, and incubation time. Initially, different concentrations (from 10 to 40 g/L) of waste carbon sources (waste baklava sherbet, banana peel powder, and peanut shell powder) were tested. The highest PHA production (OD_235_ 1.55) was observed in banana peel powder at 30 g/L concentration, and the lowest PHA production was observed in waste baklava sherbet (Fig. 2a). This decrease in PHA production may be due to the high sugar content in baklava sherbet inhibiting bacterial growth. C to N ratio is a very important factor affecting the efficiency of PHA production. Many studies reported that a high C to N ratio positively affects PHA production (Dash et al. 2020; Zhao et al. 2021). While the C to N ratio of peanut shells is higher than the banana peels, higher PHA production was achieved with banana peel powder. This situation can be explained as the high starch content of banana peels enhanced the production of PHA. Temperature plays a crucial role in PHA production by changing the physiology and diversity of microbial flora. When the results were analyzed, it was observed that the NGB15 produced PHA in a wide temperature range (15–35 °C) and the best PHA production (OD_235_ 1.73) was found to be at 25 °C (Fig. 2b). PHA production efficiency was found to be low at the high temperature (> 40 °C). This is probably due to the decreased activity of enzymes involved in PHA biosynthesis at these temperatures. Because high temperature reduces the metabolic activity (enzyme activity) of microorganisms, it could create a change in PHA content or reduce PHA production efficiency (Mahato et al. 2021).

pH is also known to affect PHA production in microbes. As shown in Fig. 2c, the maximum PHA biosynthesis was at pH 8 (OD_235_ 1.87). Alkaline pH (7–10) favored more PHA accumulation than acidic pH (4–7). Many studies reported that high pH was more suitable for PHA production. For example, Jau et al. reported that the best PHA production was achieved at pH 9 by *Spirulina platensis* (Jau et al. 2005). In another study, it was shown that biosynthesis of PHA production was observed at pH 8 (Ansari and Fatma 2016).

Also, PHA biosynthesis by strain NGB15 was tested from 1 to 7 days. Maximum PHA accumulation was observed on the fourth day (OD_235_ 1.99) (Fig. 2d). A gradual decrease in PHA production was observed after the fourth day. This decrease is probably due to the use of the produced PHA as a carbon and energy source in the following incubation periods.

After the optimization studies, optimal culture conditions were determined as 30 g/L banana peel powder, 25 °C temperature, pH 8, and 4-day incubation time. Under optimized conditions, 3.34 g/L PHA was produced. It is known that under certain conditions, *Pseudomonas* can produce PHA and store it intracellularly in inclusion bodies. Therefore, there are many studies on PHA production with *Pseudomonas* and other strains (Table 4).

**Table 4:** Microorganism and different substrates used for the production of PHA

Bacterial strain	Substrate	PHA production (g/L)	References
*P. putida*	Glucose	3.98	(Qin et al. 2022)
*P. putida*	Lignin	1.4	(Zong et al. 2022)
*P. aeruginosa*	Soybean oil	0.98	(Abid et al. 2016)
*Ensifer* sp.	Potato dextrose broth	2.75	(Khamkong et al. 2022)
*Ralstonia eutropha* 5119	Acetate	2.34	(Bhatia et al. 2019)
*P. aeruginosa* EO1	Groundnut oil	5.9	(Mahato et al. 2021)
*P. chlororaphis*	Glycerol	2.23	(de Meneses et al. 2020)
*Cupriavidus necator* and* P. citronellolis*	Apple pulp waste	3.03	(Rebocho et al. 2020)
*Bacillus* spp.	Banana peel	2.1	(Getachew and Woldesenbet 2016)
*Pseudomonas* sp.	Molasses	-	(Chaudhry et al. 2011)
*P. neustonica NGB15*	Banana peel powder	3.34	This study

Mahato et al. achieved 5.9 g/L production with *P. aeruginosa from* groundnut oil, and in another study conducted by Qin et al., 3.98 g/L PHA production was performed with *P. putida* from glucose. These amounts are higher than our production. However, in this study, a more cost-effective production was achieved by using waste materials instead of glucose. Since production cost is the most important disadvantage of bioplastics, cost-effective production is an important point.

### Characterization of PHA

#### FTIR analysis

**Fig. 3:**
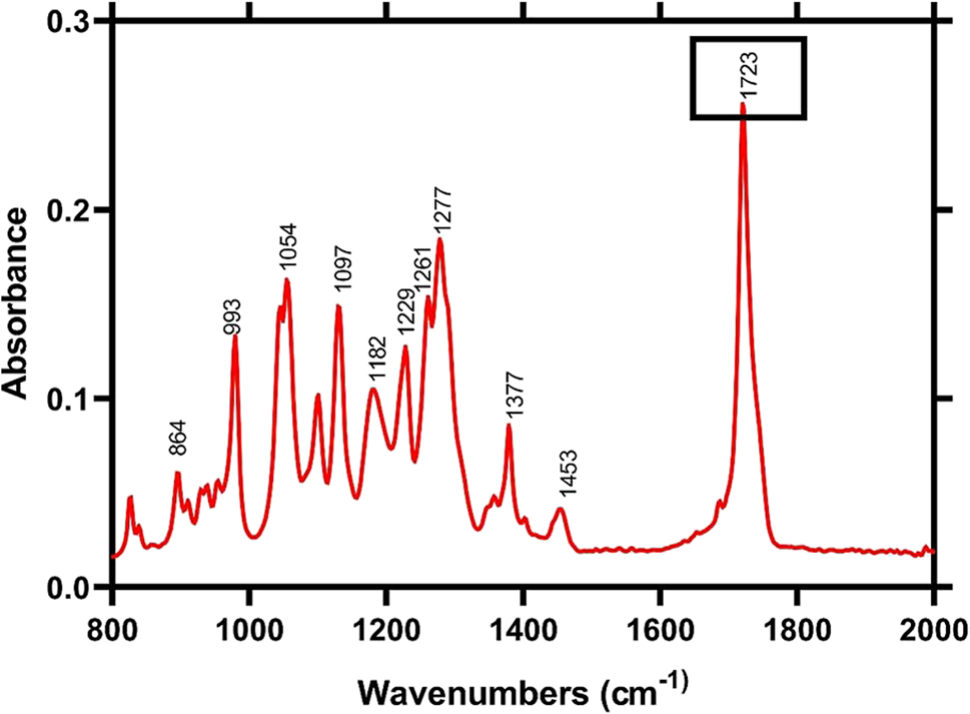
FTIR spectrum of NGB15-PHB

PHAs often have a similar formula with different R-hydroxyalkanoic acid groups attached and classified into three groups: short chain length (scl), medium chain length (mcl), and long chain length (lcl) depending on the structure, number of carbon atoms, and branching of the chain. FTIR analyses were performed to characterize the produced PHA and determine which chemical groups it contains (Fig. 3). As seen in Fig. 3, Major peaks were observed at wave numbers 1723, 1277, 1261,1097, 1054, and 993. According to these results, it was detected that the PHA produced by the NGB15 was short chain length (scl). In the literature, it was reported that a peak at 1720–1740 cm^−1^ represents the presence of an ester group (Bhagowati et al. 2015; Özgören et al. 2018), The peak at approximately 1280 cm^−1^ shows C–O–C stretching, and approximately 1263 cm^−1^ corresponds both C–O–C stretching and CH deformation. Also, 1228 cm^−1^ indicates the helical structure of the polymer. The peaks at almost 1185 cm^−1^ and 1130 cm^−1^ are determinative of asymmetric and symmetric vibrations of the C–O–C group. In addition, bands at approximately 1100 cm^−1^ and 1042 cm^−1^ show C–O–C stretching and C-CH3 stretching, respectively (Özgören et al. 2018). When these results were compared with the literature, it was determined that the type of PHA produced by NGB15 was poly-β-hydroxybutyrate (PHB) (Table 5).

**Table 5:** Comparation of the main FTIR peaks of NGB15-PHB with other PHB

Characteristic peaks (cm^−1^)			
NGB15-PHB	Commercial PHB*	*Bacillus marmarensis* -PHB*	Description
1723	1720	1720	C = O stretching
1453	1457	1454	CH_3_ asymmetric deformation
1377	1379	1378	CH symmetric deformation
1277	1280	1277	C–O–C stretching
1261	1263	1260	C–O–C stretching + CH deformation
1229	1228	1228	C–O–C stretching
1182	1178	1180	C–O–C stretching
1097	1097	1100	C–O–C stretching
1054	1044	1044	C-CH_3_ stretching

*Data taken from Özgören et al. (2018)

### Detection of the metabolic pathway of NGB15

**Fig. 4:**
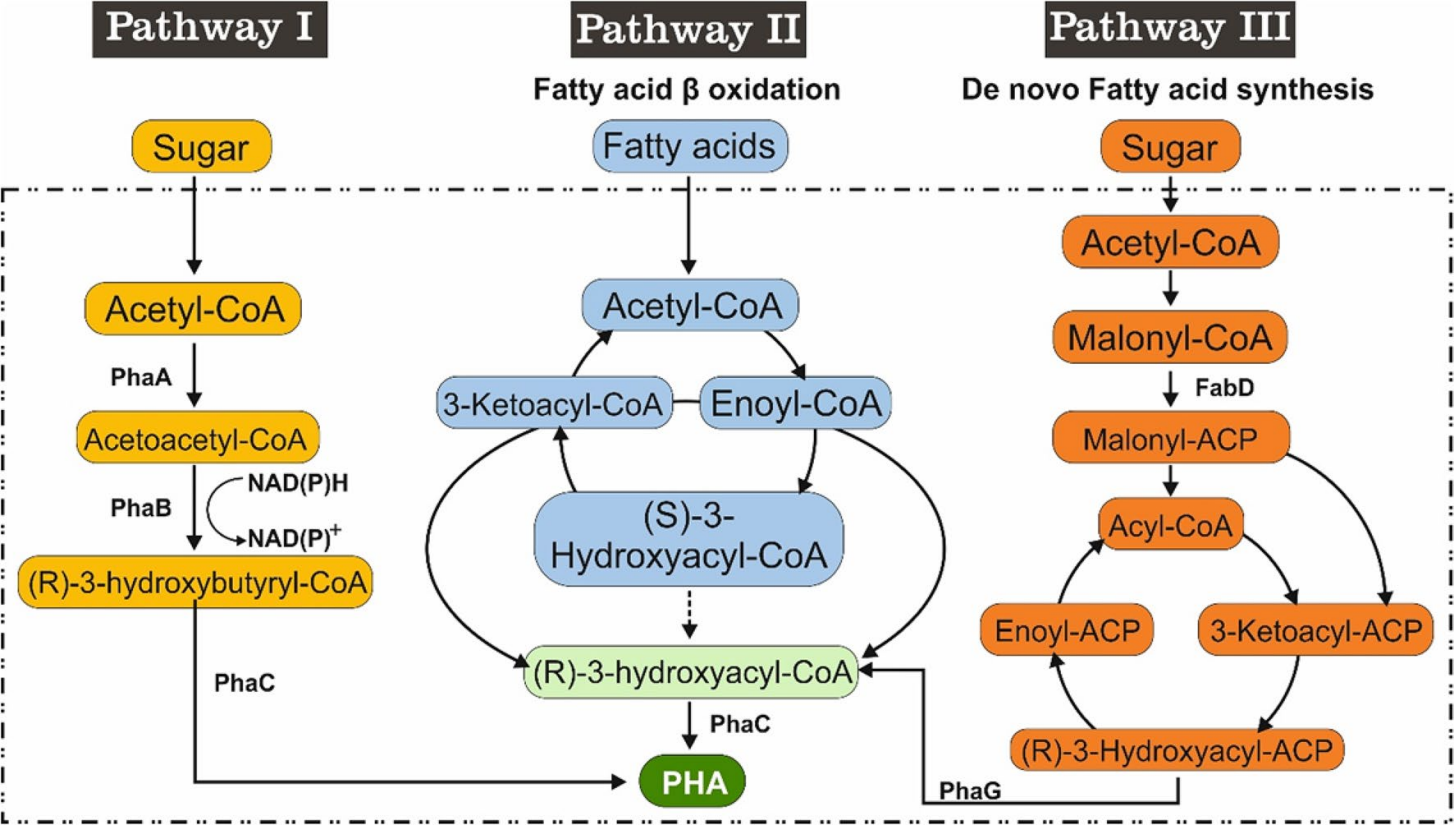
The three main metabolic pathways for PHA biosynthesis. PhaA is β-ketothiolase; PhaB is acetoacetyl coenzyme A(CoA) reductase: PhaC is PHA synthase; FabG is 3-ketoacyl acyl carrier protein (ACP) reductase; PhaG is acyl-ACP-CoA transacylase; PhaJ is enoyl-C

It has been reported that microbial PHA synthesis has three different metabolic pathways (Khatami et al. 2021) (Figure 4).

**Fig. 5:**
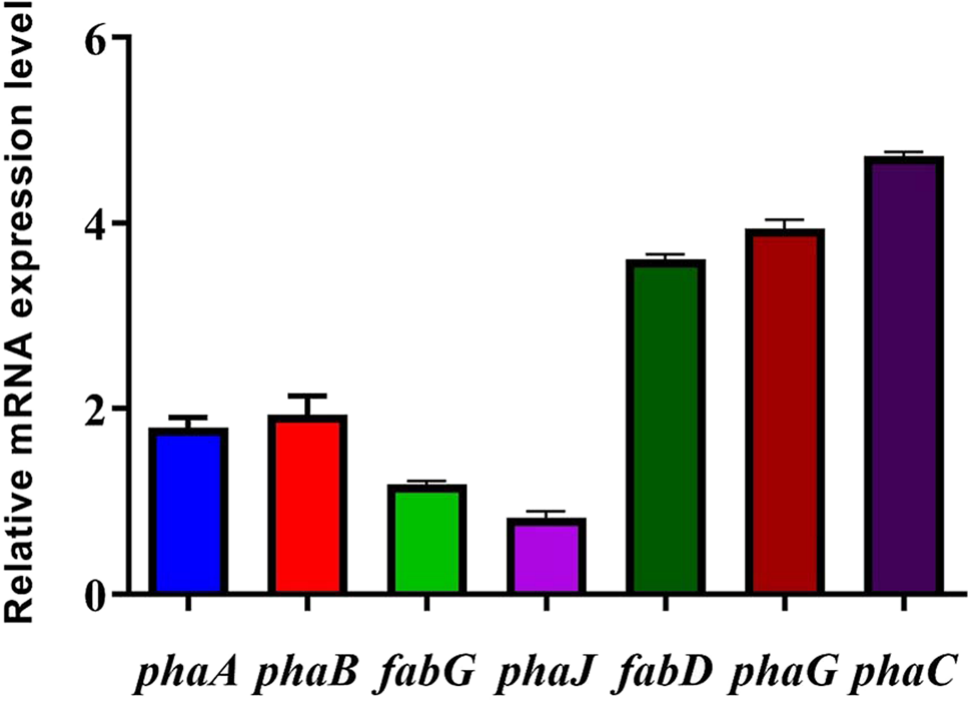
Expression profiles of key genes involved in microbial PHA synthesis

To determine which metabolic pathway NGB15 used to produce PHA, the expression of genes playing a role in these pathways was analyzed. The highest expression was observed in *fabD*, *phaG,* and *phaC* genes. According to these results, it was determined that *P. neustonica* NGB15 produces PHA using the de novo fatty acid synthesis metabolic pathway (Pathway III) (Fig. 5). In a study with *P. putida* KT2440, it was reported that PHA production was performed using the de novo fatty acid synthesis metabolic pathway (Możejko-Ciesielska and Mostek 2019b).

## Conclusion

Environmental pollution caused by conventional petroleum-based plastics has reached its peak. Therefore, with the recent projected reductions in fossil fuel resources, stringent regulations, and public awareness, the search for biodegradable and sustainable plastics is encouraging. Microbial polyhydroxyalkanoates (PHAs) are prime candidates for “green” alternatives to petroleum-based plastics. However, PHAs need to be produced at more affordable costs. The use of agricultural waste as a carbon source contributes to the preparation of a low-cost medium for PHA production. In this study, PHA production was achieved for the first time with the *P. neustonica* NGB15 using agricultural waste. Then produced PHA was characterized as poly-β-hydroxybutyrate (PHB). PHB production by *P. neustonica* NGB15 using a low-cost fermentation medium has been shown to be biotechnologically promising.

## Data Availability

The datasets generated during and/or analyzed during the current study are available from the corresponding author on reasonable request.
